# Dermatofibrosarcoma Protuberans: The Impact of the Surgical Incision Site in Relation to Tumor Recurrence

**DOI:** 10.7759/cureus.75591

**Published:** 2024-12-12

**Authors:** Bryan Felix, Suma Kaza

**Affiliations:** 1 Medicine, Avalon University School of Medicine, Willemstad, CUW; 2 Pathology, Avalon University School of Medicine, Willemstad, CUW

**Keywords:** dfsp, head and neck malignancies, incision site, mass resection, recurrent tumour, surgery, tumor removal

## Abstract

Dermatofibrosarcoma protuberans (DFSP) is a rare, locally invasive cutaneous sarcoma with a high propensity for recurrence, even following complete surgical excision. DFSP exhibits a low metastatic potential and is characterized by a distinctive honeycomb-like architecture composed of uniformly arranged spindle cells that frequently show CD34 immunostaining. Common surgical approaches include wide local excision (WLE), Mohs micrographic surgery (MMS), and, in severe cases, amputation. This literature review explores the impact of the surgical incision site on DFSP recurrence, placing particular emphasis on anatomically challenging regions, such as the head and neck, where achieving tumor-free margins is often difficult due to complex structures and limited tissue for resection. Our primary hypothesis is that DFSP cases arising in these intricate anatomical areas exhibit a higher recurrence risk compared to those on the trunk or extremities, where broader margins are more feasible. To investigate this hypothesis, data from a range of peer-reviewed studies and case reports were analyzed, including diverse patient populations from international sources, institutional case series, and large-scale database analyses, such as the Surveillance, Epidemiology, and End Results (SEER) Program. We evaluated recurrence rates, the adequacy of surgical margins, and anatomical influences across these studies while also focusing on histopathological findings like the presence of fibrosarcomatous (FS) variants, which are known to correlate with aggressive behavior and recurrence. We also reviewed emerging targeted therapies, particularly imatinib, as promising options for managing cases of unresectable or recurrent DFSP, thereby expanding therapeutic choices for clinicians when surgery alone proves inadequate. Our findings suggest a marked increase in recurrence risk for DFSP cases located in the head and neck region, attributed to limitations in achieving wide excision margins in these areas. This review underscores the importance of detailed preoperative planning, precise excision strategies, and individualized approaches based on tumor location to enhance surgical outcomes. Long-term surveillance remains crucial in DFSP management, particularly in high-risk locations, and continued research into targeted therapies offers hope for reducing recurrence rates and improving the quality of life for affected patients.

## Introduction and background

Introduction

Dermatofibrosarcoma protuberans (DFSP) is a slow-growing, locally aggressive soft-tissue sarcoma that arises in the dermis, characterized by a low likelihood of distant metastasis but with significant potential for local recurrence affecting up to 60% of patients. Although DFSP is classified within the broader family of sarcomas, it presents distinct clinical behavior, particularly in comparison to other high-grade soft-tissue sarcomas, due to its rarity and low metastatic potential [1]. The local invasiveness of DFSP, however, poses a challenge, as it often extends beyond clinically visible borders, infiltrating surrounding structures in a manner similar to more aggressive sarcomas.

Macroscopically, DFSP typically manifests as a firm, slow-growing plaque or nodule, often displaying a pink or violaceous color that may gradually increase in size. Histologically, DFSP is composed of uniformly arranged spindle cells in a storiform or honeycomb-like pattern, interwoven with fibrous tissue. A defining characteristic of DFSP is its immunoreactivity to CD34, aiding in differential diagnosis from other cutaneous or subcutaneous lesions [2]. Microscopic examination further reveals that DFSP commonly extends deeper than the visible lesion, necessitating careful surgical planning to achieve clear margins [1,3]. DFSP does not have any associated risk factors and can arise on healthy skin as well as chronically damaged areas. It has an incidence of three to five cases per million population each year [3]. 

Clinically, DFSP is sometimes misidentified as benign growths such as dermatofibromas, keloids, or nodular fasciitis. This is due to the overlapping fibroblastic characteristics and similar presentations [4]. The tumor most commonly appears on the trunk and upper limbs, with fewer cases occurring in the head and neck regions, where surgical margin challenges can increase the risk of recurrence. Lesions in the head and neck regions carry a higher recurrence risk, likely due to challenges in achieving adequate surgical margins in these anatomically constrained areas [1]. This underscores the need for a coordinated, multidisciplinary treatment approach involving dermatologists, oncologists, and surgeons to optimize management and improve patient outcomes.

Search stratergy

A comprehensive literature search was conducted to identify studies discussing DFSP, with a specific focus on recurrence, treatment outcomes, and management strategies. The search spanned PubMed, Embase, and Cochrane Library, covering articles published between January 2000 and December 2023. The search strategy incorporated MeSH terms and keywords such as “dermatofibrosarcoma protuberans,” “DFSP,” “recurrence,” “treatment outcomes,” “wide local excision,” “Mohs micrographic surgery,” “fibrosarcomatous transformation,” and “systemic therapy.” Only English-language studies involving human subjects and directly addressing DFSP recurrence or treatment outcomes were included. Peer-reviewed case reports, retrospective cohort studies, randomized controlled trials, and systematic reviews were eligible for inclusion. In contrast, non-English language articles, non-human studies, and publications unrelated to DFSP recurrence or treatment outcomes were excluded. Additionally, abstracts, editorials, and letters to the editor that lacked original data were not considered.

## Review

Clinical presentation and diagnosis

Patients often present with a lesion displaying a violet or pink coloration, exhibiting either slow growth or a stable size. Dermatofibrosarcoma protuberans primarily manifests in the dermis and subcutis of the trunk (50-60%) and upper limbs (25%), with the head and neck being affected in 10-15% of cases [5]. The majority of lesions are located in the proximal extremities; however, DFSP has also been reported to occur in the distal extremities and acral regions.

Dermatofibrosarcoma protuberans usually follows an asymmetric growth pattern along with finger-like projections with lateral or deep extension varying from 0.3 cm to 12 cm over the macroscopic borders [2]. If left untreated, DFSP can locally invade more deeply into the fascia, muscle, periosteum, and bone and, occasionally but rarely, metastasize to the lung, brain, bone, visceral organs, soft tissues, and lymph nodes [6].

Deep subcutaneous punch biopsy or incisional biopsy remains the gold standard for diagnosis [7]. The characteristic histological features include monomorphous spindle cell proliferation, forming a honeycomb appearance [7]. Immunohistochemical studies can further support the diagnosis, with the tumor cells typically expressing CD34, which is a marker of fibroblastic differentiation. The immunohistochemical evaluation demonstrated the expression of vimentin, CD34, apolipoprotein D, and nestin in tumor cells. Additionally, weak or inconsistent staining for EMA was observed. However, tumor cells were negative for desmin, S100 protein, FXIIIa, stromelysin III, HMGA1 & 2, tenascin, D2-40, CD163, and keratins [7].

To differentiate DFSP from other benign and malignant neoplasms, such as dermatofibroma, schwannoma, cutaneous neurofibroma, solitary fibrous tumor, intradermal spindle cell lipoma, and spindle cell or desmoplastic melanoma, a thorough evaluation of clinical manifestations, histopathological features, and CD34 immunostaining profiles is imperative [6]. In addition to histopathology, imaging studies also play an important role in the evaluation of DFSP. HR-MRI (high-resolution magnetic resonance imaging) and CT (computed tomography) are particularly useful in evaluating the extent of cutaneous involvement and delineating tumor margins [4]. These recent advancements in imaging have improved the precision of preoperative planning by enhancing visualization of DFSP tumor boundaries, particularly in complex regions like the head and neck. Overall, a multimodal diagnostic approach, including clinical examination, histopathological assessment, and imaging studies, is necessary for the accurate diagnosis as well as management of DFSP.

Prognosis

Dermatofibrosarcoma protuberans originates from the dermis or subcutis and spreads horizontally as well as vertically, which results in the destruction of surrounding structures. It is due to this infiltrative and unfavorable growth pattern that local recurrences are relatively common [8].

Insights from the Surveillance, Epidemiology, and End Result (SEER) Program - a large retrospective analysis encompassing 3,686 DFSP cases diagnosed from 1972 to 2012 - highlight several prognostic factors, notably age, male sex, and tumor size, as significant determinants of recurrence risk and overall prognosis [8,9]. Advanced age has been correlated with higher recurrence rates in DFSP. This trend may be attributed to age-associated declines in immune function, which could impair the body’s ability to contain tumor growth and limit its invasive potential. Moreover, older patients often present with larger tumors, which may be due to delayed detection and prolonged undiagnosed growth, thus increasing the complexity of surgical intervention [8]. Male sex has also been linked with an elevated risk of recurrence, though the exact biological mechanisms remain uncertain. Hormonal influences, genetic factors, or delays in seeking treatment may play a role, while disparities in healthcare access between men and women have also been considered as possible contributing factors [9]. Tumor size is another critical prognostic factor, with larger tumors often posing challenges in surgical resection. These tumors tend to invade deeper tissue layers and are more likely to have microscopic residual disease, especially in complex anatomical sites like the head and neck. Larger tumor size generally indicates a longer period of unmitigated growth, underscoring the importance of prompt detection and extensive resection margins [9].

These findings underscore the importance of factoring in patient age, sex, and tumor size when strategizing treatment, allowing for individualized management approaches and reinforcing the need for long-term surveillance to detect recurrences early.

Pathogenesis

DFSP is primarily driven by a specific chromosomal translocation, t(17;22)(q22;q13), which results in the formation of the COL1A1-PDGFB fusion gene. This genetic abnormality leads to the continuous production of platelet-derived growth factor beta (PDGFB), causing autocrine activation of PDGFB receptors and promoting cellular proliferation and survival in the tumor cells. The overexpression of this growth factor contributes to DFSP's locally invasive nature by facilitating an environment conducive to cell proliferation and resistance to apoptosis [8,10]. While over 34 variants of this fusion gene have been identified, two notable subtypes predominate: the Bednar pigment variant, accounting for less than 5% of cases, and the higher-grade fibrosarcomatous variant, representing 5-15% of the patient population.

To detect the t(17;22) translocation and other associated transcripts, techniques such as fluorescence in situ hybridization (FISH) and reverse transcription polymerase chain reaction (RT-PCR) are utilized. These methods provide sensitive and specific tools for diagnosing DFSP, allowing for the identification of the COL1A1-PDGFB fusion gene and facilitating timely and accurate treatment decisions [11]. These alterations may further enhance the aggressive behavior of the tumor and its potential for recurrence, as they contribute to uncontrolled cell growth and evasion of apoptosis [11].

The understanding of these molecular mechanisms has led to the development of targeted therapies, such as imatinib, a tyrosine kinase inhibitor that effectively inhibits PDGFB receptor phosphorylation. This targeted therapy exemplifies how a molecular understanding of the disease can lead to tailored treatments that improve patient outcomes, particularly in cases where traditional surgical excision may be insufficient or not feasible [2].

Treatment

The cornerstone of treatment for DFSP is wide local excision (WLE) with a 2-3 cm tumor-free margin, encompassing the skin, subcutaneous tissue, and underlying fascia in a three-dimensional resection [12]. Increasing the surgical margin to 3 cm has been shown to significantly reduce recurrence rates, and further increasing the margin to 5 cm resulted in no reported recurrences following surgery [13]. While wider margins are effective in minimizing recurrence, they may lead to larger tissue defects that necessitate extensive reconstruction, which could impact the patient’s quality of life.

An alternative to wide local excision is Mohs micrographic surgery (MMS), which enables the clinician to examine 100% of the tumor margins, identify microscopic extensions, and excise them while preserving healthy tissue [2,14]. According to Paradisi et al., significantly lower recurrence rates were recorded in patients subjected to MMS compared to those treated with WLE [5]. However, the disadvantages of MMS are that the tumor cells can be confused with normal spindle cells of the dermis, and CD34 staining of frozen sections has high variability [2]. The management of dermatofibrosarcoma protuberans is complex, time-consuming, and costly, with a paucity of randomized controlled trials and comparative studies [2]. Radiation therapy can also be integrated with resection when wide surgical margins are not possible. It can also be used as adjuvant therapy after resection in case of recurrence [2]. Table 1 enumerates the pros and cons of wide local excision and Mohs micrographic surgery.

**Table 1:** Comparison of surgical approaches for the treatment of DFSP: pros and cons WLE: wide local excision; MMS: Mohs micrographic surgery; DFSP: dermatofibrosarcoma protuberans [2,5].

Surgical method	Pros	Cons
WLE	Allows for a larger excision	Potential for larger defects requiring reconstruction
			May require wider margins, leading to more extensive surgery
		Simple and quicker procedure	
	Higher recurrence rate	
			MMS	Offers precise removal of cancerous tissue while preserving healthy tissue	Longer and more complex procedure
	Lower recurrence rate	Higher cost	
Real-time examination of the margins reduces the chance of incomplete excision	May require multiple stages of surgery		

Imatinib has also been shown to be effective in patients with the t(17;22) positive translocation but lacks against the t(17;22) negative translocation [2]. Imatinib inhibits tyrosine kinase and may be able to induce tumor regression in patients with recurrent DFSP, unresectable DFSP, or metastatic DFSP [15]. Prior to initiating imatinib therapy, it is recommended to detect the COL1A1-PDGFB fusion translocation using FISH or reverse transcriptase-polymerase chain reaction (RT-PCR). Sunitinib malate, a potent inhibitor of multiple tyrosine kinases, can be employed as a salvage therapy for imatinib-refractory neoplasms [16].

Recurrence of DFSP 

Recurrence rates as high as 50% have been described in literature. Recurrence is most common for tumors of the head and neck, likely because it is difficult to achieve wide margins in these areas [2]. A wider surgical margin, with a minimum of 2 cm, can potentially reduce the recurrence rate of head and neck dermatofibrosarcoma protuberans (HNDFSP). However, the decision to pursue a wider margin should be made carefully, taking into account the potential reconstructive, functional, and aesthetic consequences [17]. The recurrence rate is notably higher for DFSP-FS (fibrosarcoma change) compared to classic DFSP. This increased recurrence can be attributed to the aggressive nature of fibrosarcomatous variants, which often exhibit a more infiltrative growth pattern and may possess distinct molecular characteristics, including alterations in genetic markers that promote tumor aggression. These differences can result in a higher likelihood of residual disease after surgical excision. Most recurrences for DFSP-FS develop within three years of the initial excision, underscoring the importance of vigilant follow-up in these patients [18]. Misdiagnosis is another common cause of recurrence because the risk of recurrence becomes greater with the increase in the number of treatments [19]. A systematic review and meta-analysis that included multiple databases, such as Ovid MEDLINE (1946-2018), Embase (1988-2018), Web of Science (1975-2018), and Scopus (1970-2018), analyzed a variety of studies focusing on the efficacy of MMS versus WLE in managing DFSP. This comprehensive approach allowed for a robust comparison of recurrence rates across diverse patient populations and treatment settings. The findings indicated that the local recurrence rate of DFSP in patients treated with MMS is lower than in those treated with WLE, suggesting that MMS should strongly be considered when available [20]. Another meta-analysis conducted on March 6, 2023, concluded that MMS has a survival advantage in recurrent tumors [20]. Table 2 enumerates the predisposing factors associated with the recurrence of this neoplasm.

**Table 2:** Risk factors for DFSP recurrences DFSP-FS: dermatofibrosarcoma protuberans fibrosarcoma change.

Risk factors for DFSP recurrences [21]
DFSP-FS
<1 mm to positive microscopic margins
Increased cellularity
High mitotic index
Age > 50

Dermatologists and surgeons should be cognizant of the high recurrence rate associated with DFSP, even with wide local excisions and extensive margins. Consequently, long-term postoperative surveillance is essential for these patients.

Impact of incision site

Approximately half of all DFSP cases are located on the trunk, followed by the extremities, which account for 30-35% of cases. The head and neck region, however, is less frequently involved, comprising approximately 10-15% of all cases [17,22]. The head and neck region has been documented to exhibit the highest rate of local recurrence following local excision, reaching up to 56% [22,23].

A long-term follow-up of patients with HNDFSP was conducted at Shanghai Ninth People’s Hospital, concluding that HNDFSP is significantly challenging to control locally and carries a worse prognosis with current treatment strategies [24]. Recent studies have also shown that it is much more invasive than DFSP of the trunk. Numerous reports of metastasis to the lung, which is rare, have also been documented [17]. One case reported a patient with DFSP in the head and neck region who experienced a local recurrence just two months after initial surgery [25]. A study conducted by Mani et al. revealed a 53% incidence of local recurrence in 17 cases of head and neck dermatofibrosarcoma protuberans [17,24]. Mark et al. also reported a very high local recurrence rate of 60% in a series of 16 patients suffering from DFSP in the head and neck region [26]. A retrospective analysis of individuals with DFSP of the head and neck region, conducted between 2016 and 2021, revealed that three of three patients who underwent resection experienced tumor recurrence. Two of these patients had received primary treatment elsewhere [17]. In addition, a study by Farma et al. involving 204 cases of dermatofibrosarcoma protuberans identified only two recurrences, both of which occurred in the head and neck region [27]. 

These observations raise the possibility that lesions in the head and neck area might be more prone to recurrence than in other anatomic sites.

## Conclusions

Dermatofibrosarcoma protuberans is a cutaneous malignancy characterized by local invasiveness and a notable propensity for recurrence, even after seemingly complete tumor eradication. Various unfavorable prognostic factors contribute to the likelihood of relapse, including age over 50 years, high mitotic rates, and increased cellularity. Tumors located in the head and neck region are particularly prone to recurrence, although the underlying reasons remain unclear. One potential explanation is that the intricate anatomy and limited surgical access in these areas complicate tumor excision. Additionally, the site of the surgical incision may also influence recurrence, as incisions in certain regions might leave residual tumor cells or affect healing, ultimately leading to increased recurrence rates.

To effectively manage DFSP, an aggressive multidisciplinary approach is essential. The primary goal in treating patients with DFSP is to achieve complete surgical clearance. Due to the malignancy's notorious tendency to recur, long-term follow-up and periodic observation are crucial for early detection of any recurrence. Future research should focus on investigating the molecular pathways associated with DFSP recurrence, including the role of genetic and epigenetic factors, as well as developing targeted therapies that could improve patient outcomes. Additionally, exploring the impact of surgical techniques and incision sites on recurrence rates may yield valuable insights that enhance treatment strategies. Overall, a comprehensive understanding of the factors influencing DFSP recurrence will be vital for advancing the management and therapeutic approaches for this challenging malignancy.
